# Screening of Gestational Diabetes and Its Risk Factors: Pregnancy Outcome of Women with Gestational Diabetes Risk Factors According to Glycose Tolerance Test Results

**DOI:** 10.3390/jcm11174953

**Published:** 2022-08-23

**Authors:** Ele Hanson, Inge Ringmets, Anne Kirss, Maris Laan, Kristiina Rull

**Affiliations:** 1 Department of Obstetrics and Gynaecology, Institute of Clinical Medicine, University of Tartu, Puusepa St. 8, 50406 Tartu, Estonia; 2 Women’s Clinic, Tartu University Hospital, Puusepa St. 8, 50406 Tartu, Estonia; 3 Institute of Family Medicine and Public Health, University of Tartu, Ravila St. 19, 50411 Tartu, Estonia; 4 Institute of Biomedicine and Translational Medicine, University of Tartu, Ravila St. 19, 50411 Tartu, Estonia

**Keywords:** gestational diabetes, screening test, hyperglycemia, macrosomia, oral glycose tolerance test

## Abstract

*Background*: Gestational diabetes mellitus (GDM) can cause maternal and neonatal health problems, and its prevalence is increasing worldwide. We assessed the screening of GDM during a 7-year period and compared the outcome of pregnancies at high risk for GDM. *Methods*: We analyzed non-selected pregnant women (*n* = 5021) receiving antenatal care in Tartu University Hospital, Estonia in 2012–2018. Pregnant women were classified based on the absence or presence of GDM risk factors as low risk (*n* = 2302) or high risk for GDM (*n* = 2719), respectively. The latter were divided into subgroups after the oral glycose tolerance test (OGTT): GDM (*n* = 423), normal result (*n* = 1357) and not tested (*n* = 939). *Results*: The proportion of women with GDM risk factors increased from 43.5% in 2012 to 57.8% in 2018, and the diagnosis of GDM more than doubled (5.2% vs. 13.7%). Pregnancies predisposed to GDM but with normal OGTT results were accompanied by an excessive gestational weight gain and increased odds to deliver a LGA baby (AOR 2.3 (CI 1.8–3.0)). *Conclusions*: An increasing number of pregnancies presenting GDM risk factors are diagnosed with GDM. Pregnant women with GDM risk factors are, despite normal OGTT, at risk of increased weight gain and LGA newborns.

## 1. Introduction

Gestational diabetes mellitus (GDM), glycose tolerance disorder with onset or first recognition during pregnancy, is the most frequent complication of gestation. According to a report from the International Diabetes Federation, every 6th birth was complicated by GDM in 2019 [1].

GDM increases the risk of delivering a large for gestational age newborn (LGA) and related complications such as operative delivery, lacerations in the birth canal, birth trauma and the poor adaptation of the newborn [2]. Additionally, impaired glucose metabolism during the pregnancy is associated with preeclampsia and premature delivery [3,4]. In the long term, approximately half of women with GDM will develop type 2 diabetes later in life [5]. Therefore, screening, early initiation of counselling and treatment remains crucial [6].

Currently there is no generally accepted screening group or “gold standard” test to define the disease status. In most centers, the oral glucose tolerance test (OGTT) is applied but the testing strategy and diagnostic criteria vary [7,8]. In 2010, the International Association of Diabetes and Pregnancy Study Groups (IADPSG) recommended the use of 75 g OGTT at 24–28 gestational weeks with a cut-off point of fasting venous plasma glucose ≥5.1 mmol/L and/or after 1 h and 2 h level of ≥10.0 mmol/L and ≥8.5 mmol/L, respectively [9]. The recommendations are based on the results of the Hyperglycemia and Adverse Pregnancy Outcome (HAPO) study, which demonstrated a continuous relationship between maternal hyperglycemia and adverse perinatal outcome [3].

As in many countries, in Estonia, the decision of OGTT referral is based on the presence of GDM risk factors [10]. The most commonly recognized risk factors for GDM are obesity (BMI >30 kg/m^2^), GDM and/or of birth baby >4500 g during any of the previous pregnancies, diabetes mellitus (DM) among first-degree relatives, ethnicity with a high prevalence of diabetes and previous polycystic ovary syndrome (PCOS) [10,11].

After the diagnosis of hyperglycemia, a pregnant woman is referred to diet counselling and if the blood glucose level exceeds the target, metformin and/or insulin, are administrated. Treatment of GDM, lifestyle changes and timing of delivery have shown to reduce serious perinatal complications such as the rate of macrosomia and the long-term consequences of GDM [12,13].

The pregnancy outcome of women presenting GDM risk factors who skip the OGTT or whose glycose levels remain below the GDM diagnostic cutoff and therefore continue usual follow-up without additional dietary restrictions is addressed less. High risk untested pregnant women include those with possible undiagnosed GDM and prone to poor gestational outcome [14].

The aim of this study is to assess the prevalence of GDM and its risk factors in 2012–2018 in Estonia and to compare the outcome of pregnancies predisposed to GDM in cases with and without subsequent GDM diagnosis.

## 2. Materials and Methods

### 2.1. Study Population

We performed a retrospective observational study including 5735 pregnant women receiving antenatal care in Women’s Clinic Tartu University Hospital (TUH), Estonia in 2012–2018.

The study participants were recruited during three time periods. The first study cohort was compiled to assess the compliance to the GDM screening algorithm, and it incorporated data of all women starting antenatal follow-up visits between January and December 2012 (*n* = 1373) [15]. The second set of women (*n* = 2334) originated from a monocentric prospective “Happy Pregnancy” (HP) study (full name “Development of novel non-invasive biomarkers for fertility and healthy pregnancy”: principial investigator Prof. Maris Laan). The recruited women included approximately two thirds of unselected pregnant women receiving antenatal care in TUH between March 2013 and August 2015 [16,17,18]. The third dataset comprised all women whose antenatal follow-up in TUH started between January and December 2018 (*n* = 2028).

The considered GDM risk factors included pre-pregnancy overweight/obesity (BMI 25–29.9/>30 kg/m^2^), GDM and/or of birth newborn >4500 g during any of the previous pregnancies, DM among first-degree relatives, PCOS, fasting glucose >5.1 mmol/L, glycosuria, excessive weight gain, suspicion of a LGA fetus or polyhydramnion during the index pregnancy. The information about the GDM risk factors, course and outcome of the pregnancy was collected by midwives and/or extracted from electronic hospital medical records (Supplementary Table S1).

The women with pregestational DM including type 2 DM diagnosed at the 1st trimester (fasting plasma glycose ≥7.0 mmol/l or any plasma glycose above 11.1 mmol/L) (19), multiple pregnancy or termination of pregnancy before 22 gestational weeks (g.w.), and those who had missing delivery data were excluded from the analysis. 

The final dataset included a total of 5021 pregnancies: 1073 women represented the first (2012), 2176 women the second (2013–2015) and 1772 women the third (2018) recruitment cohort. All participants were of white European ancestry and Estonian residents.

### 2.2. Patient Grouping and Diagnostic Criteria

**Figure 1:**
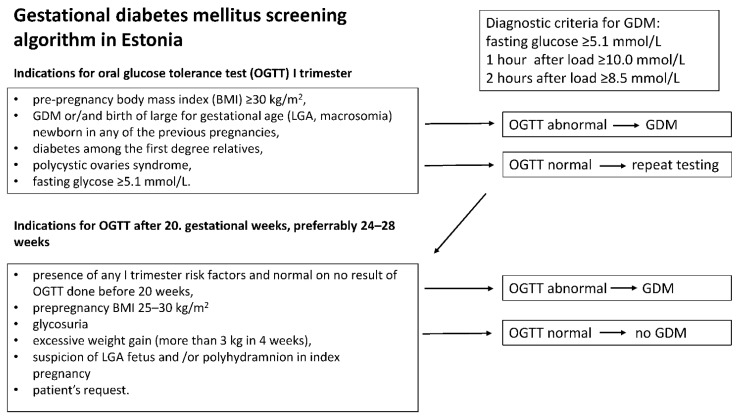
Gestational diabetes mellitus screening algorithm in Estonia.

The GDM screening algorithm applied in Estonia since 2011 is shown in Figure 1 [19]. According to the current algorithm and clinical guidelines, OGTT is not mandatory to all pregnant women. Only women presenting any GDM risk factor are referred to OGTT.

GDM was diagnosed when any of the three consecutive measurements of oral glucose tolerance test (OGTT) were abnormal. Values were considered abnormal if the fasting venous plasma glucose level was ≥5.1 mmol/L and/or 1 h or 2 h after administration of 75 g oral glucose orally resulted in plasma glucose levels of ≥10.0 mmol/l and/or ≥8.5 mmol/L glucose, respectively (19). 

Women were further categorized into four subgroups according to the presence of GDM risk factors and OGTT results: (1) low risk (group 1): women without risk factors and no indication to OGTT (*n* = 2302, 46%); (2) no OGTT (group 2): women with risk factors but no OGTT or only one normal test result before 20 weeks (*n* = 939, 19%); (3) normal OGTT (group 3): women with risk factors and a normal OGTT result obtained after 20 g.w. (*n* = 1357, 27%); (4) GDM (group 4): women with an abnormal OGTT result at any time during the gestation (*n* = 423, 8%).

For the assessment of a newborn’s weight, a growth calculator based on INTERGROWTH-21st Project [20] data was applied to convert the newborn birthweight into gestational age and sex-adjusted centiles. Large-for-gestational-age (LGA) newborns were diagnosed as birthweight ≥95th centile and small-for-gestational-age (SGA) newborns as birthweight ≤10th centile.

Birth <37th g.w. was defined as preterm birth (PTB). Gestational hypertension (GH) was diagnosed if a patient exhibited after 20 g.w. new-onset isolated hypertension (≥140 mmHg and/or ≥90 mmHg). Preeclampsia (PE) was diagnosed if a patient exhibited hypertension after 20 g.w. accompanied by any of the following new-onset conditions: proteinuria, renal insufficiency, impaired liver function; hematological or neurological complications and eclampsia [21]. Only perineal ruptures after vaginal delivery involving anal sphincter (3rd grade) and/or anal epithelium (4th grade) were analyzed.

Total weight gain during the pregnancy was considered excessive when it exceeded the widely accepted recommendations: >9.0 kg for obese (pre-pregnancy BMI 30.0 kg/m^2^ or higher); >11.5 kg for overweight (25.0–29.9 kg/m^2^), >16.0 kg for normal weight (18.5–24.9 kg/m^2^) and >18.0 kg for underweight women (less than 18.5 kg/m^2^) [22].

### 2.3. Statistical Analysis

Summary estimates of the data (median, 5th–95th centile) were calculated and all statistical tests were implemented using the STATA software ver. 13.1 (StataCorp, College Station, TX, USA). To compare groups, the Wilcoxon rank-sum test was used for continuous variables and Chi^2^ test for categorical variables. A significance level of 0.05 was used. Bonferroni correction for multiple testing was applied according to the number of tests performed. One-way ANOVA was used for continuous variables to detect the differences in multiple group comparisons. In case of significant difference for post hoc pairwise comparisons, the Wilcoxon rank-sum test was applied. Binary logistic regression analysis was used to examine the association between pregnancy outcome and allocated GDM risk group. The adjusted odds ratios with 95% confidence intervals were calculated. The odds ratios (OR) were adjusted for previous births, LGA baby, pre-pregnancy BMI, age, cohort, or gestational age at delivery depending on pregnancy outcome variable.

### 2.4. Ethical Approval

The data collection and analysis was approved by the Research Ethics Committee of the University of Tartu, Estonia (permissions no. 225/T-6, 06.05.2013; 221/T-6, 17.12.2012, 286/M-18, 15.10.2018; 291/T-3, 18.03.2019 and 322/M-17, 17.08.2020) and the study was carried out in compliance with the guidelines of the Declaration of Helsinki.

## 3. Results

### 3.1. The Prevalence of Gestational Diabetes and Its Risk Factors Has Increased during Seven Years

The study population comprised 5021 unselected pregnant women from three recruitment periods across seven years (Table 1).

Between 2012 and 2018, the proportion of pregnant women presenting any of the GDM risk factors increased from 43.5% to 57.8%. The most prevalent GDM risk factor was overweight (BMI 25–29.9 kg/m^2^; >30% of high-risk women), followed by women with a high fasting glycose level (10.3–38.3% of pregnancies) and “other” risk factors (9.5–23.9%): glycosuria, excessive weight gain and suspicion of LGA fetus. In 2018, more high-risk women were subjected to the GDM screening algorithm compared to 2012 and 2013–2015 cohorts (Table 1). In addition, compared to the first two cohorts, the women in 2018 were older: 29 (5–95th percentile 21–38) years versus 28 (20–38) years in both 2012 and 2013–2015 cohort, and had a higher BMI compared to the 2013–2015 cohort (22.7 vs. 22.3) (Table 1).

Women with obesity and/or GDM in a previous pregnancy were more frequently subjected to the correct GDM screening algorithm (75.1–88.1% of risk factor carriers) (Table 2) and were more likely to receive a GDM diagnosis: obesity (OR 6.3 (5.0–8.9)), previous GDM (OR 12.5 (95% CI 7.5–20.6)). Although women with “other” risk factors were most often tested, only 14.7% received a GDM diagnosis (Table 2).

While the proportion of LGA babies has slightly decreased since 2012 from 18.8% to 16.1% in 2018 (*p* = 3.4 × 10^−2^), it remains not statistically significant after Bonferroni correction (*p* < 7.6 × 10^−4^). Additionally, the occurrence of pregnancy complications (preterm delivery and birth of SGA babies) and C-section rate has not changed notably during the examined period (Table 1).

### 3.2. The Largest Babies Are Born to Mothers Who Undergo Correct GDM Screening Algorithm

The pregnancy outcome was assessed comparatively in the four subgroups formed based on the presence of GDM risk factors and outcome of the OGTT test.

Compared to other subgroups, mothers with GDM (group 4) had the lowest gestational age at delivery, were more likely to deliver a LGA baby and more often via C-section (Table 3 and Table 4). Gestational hypertension has also been more frequently reported among the GDM cases (Table 3).

The median birthweight of newborns was highest among women with risk factors to GDM, but a normal OGTT result (group 3) compared to other non GDM groups. Birthweight centiles were similar in women presenting risk factors to GDM and receiving OGTT irrespective of the OGTT result (~82 percentile), but significantly higher compared to those with risk factors but no OGTT (group 2) (Table 3).

Additionally, the C-section rate of low-risk women (group 1) was lower compared to women with normal OGTT (group 3) and GDM (group 4) (Table 3).

Birthweight and the proportion of LGA babies was the lowest among low-risk women (group 1) compared to all high-risk women (groups 2, 3 and 4) (Table 3). An inverse trend, however, not significant, was noted in the prevalence of SGA newborns (3% vs. ≤1.7%).

### 3.3. Comparison of Maternal Characteristics and Pregnancy Course among High-Risk Pregnant Women with Normal or No OGTT Result

Women presenting GDM risk factors but a normal OGTT result had significantly more LGA babies compared to those with no OGTT result and, therefore, their GDM status was unknown (Table 3 and Table 5). Among women presenting risk factors to GDM, the odds to deliver a LGA baby after a normal OGTT result was nearly as high as in the GDM diagnosis group (Table 4).

Both birthweight and centile of newborns were significantly lower in high-risk non-OGTT women (group 2) in contrast to the high-risk but normal OGTT group (group 3) (Table 3 and Table 5). However, in both groups approximately every fifth woman delivered by C-section; 31% of operative deliveries among women with normal OGTT (group 3) resulted in the birth of a LGA baby compared to 18% in the no OGTT subgroup (group 2).

There was no difference in maternal age and pre-pregnancy BMI between these two groups.

Although the birth of a LGA baby in any of the previous pregnancies accounted for a small number of women as a risk factor, more of them had normal OGTT (group 3). The percentage of women with “other” risk factors was considerably higher in the normal OGTT group (26.4% vs. 3.6%). However, women with increased fasting glycose level detected in the first trimester were in the no OGTT group (group 2).

There was higher total weight gain among women with a normal OGTT (group 3) result compared to non-OGTT women (group 2). Weight gain difference was observed especially after 24 g.w. when OGTT is usually scheduled. Women with normal OGTT (group 3) gained, on average, 11.6 ± 5.7 kg, median 11 kg, compared to 10.2 ± 4.6 kg, median 10 kg in the no OGTT group (group 2) (Table 5). In comparison, the total weight gain in women with GDM 12.7 ± 8.7 kg, median 11.7 kg, and after 24 gestational week 8.3 ± 5.1 kg, median 8 kg.

### 3.4. Pregnancy Course and Outcome of Women with GDM According to Treatment

In our study, out of 423 women with a GDM diagnosis, 82 (19.4%) needed medical treatment (metformin and/or insulin) in addition to dietary measures. The women receiving medication delivered earlier compared to women whose GDM was controlled with diet (274.5 vs. 277 g.d, *p* = 1.3 × 10^−3^). Apart from gestational age at delivery, we did not detect any differences in pregnancy course and outcome between different treatment modalities (Supplementary Table S2).

## 4. Discussion

We evaluated the pregnancy course and outcome of low and high-risk women with and without GDM diagnosis after the OGTT test at Women’s Clinic of Tartu University Hospital, Estonia in 2012–2018. Our study shows sharp increase in GDM risk factor carriers, almost doubling the women diagnosed with GDM over the seven-year period. Additionally, we observed a high fraction of LGA newborns among women carrying GDM risk factors but defined as unaffected based on the current GDM screening algorithm. These women also underwent excessive weight gain during the second half of pregnancy.

In Estonia, the risk factor-based testing of GDM is applied and the reported prevalence of the disease is influenced by the testing activity. Referral to OGTT is dependent on the subjective assessment of risk factors by midwives or obstetricians, as well as the patient’s consent and understanding of the necessity of the test. By 2018, more than half of pregnant women had at least one risk factor but only three of four (76.8%) received the OGTT test, as suggested by the guidelines in [19]. Those who remain untested may have undiagnosed GDM and are therefore prone to GDM-related complications, including stillbirth [23]. A study in Finland found that even mild untreated hyperglycemia resulted in an increased Cesarean section rate and larger birth weight [24].

Benhalima et al. studied selective screening for GDM in European countries and found that by using the risk factor-based screening algorithm, more than a third of GDM cases would be missed [10]. They also suggested that to improve testing, the selection of risk factors should be simplified: by screening all women at age 30 or more and/or BMI ≥ 25 kg/m^2^, 70% of pregnant women would need OGTT with missed GDM cases of 18.6% [10]. Furthermore, OGTT testing is conducted between 20 and 30 g.w., adding additional OGTT after that period could help to determine late onset GDM with increased risk of operative delivery. Sasson et al. found that pathological OGTT at term due to the suspicion of LGA resulted in a higher rate of Cesarean section [25].

Another option to improve GDM diagnostics would be universal screening, ensuring that every woman is at least offered testing. Universal testing would result in the maximum number of GDM cases at the expense of increased healthcare costs and the workload of clinical personnel; however, overall, this tends to be cost-effective [26]. As GDM also bears responsibility for long-term complications, mothers and offspring would benefit from universal screening and lifestyle interventions when considering their health risks in later life. [27,28,29].

The aim of detecting most GDM cases would be lowering the risk of complications after successful intervention. The most frequent complication of poorly controlled GDM is a birth of a LGA neonate [3]. In addition to the GDM group, we could expect larger newborns in a high-risk group who have skipped OGTT, possibly due to undiagnosed GDM. However, our data showed a comparable number of LGA neonates between the GDM group and women with a normal OGTT result. This could be explained by the fact that GDM is not the only factor resulting in fetal macrosomia; other known risk factors are multiparity, older age, previous LGA and a male newborn. In addition, pregnancy weight gain and pre-pregnancy BMI have been shown in previous studies to be related to GDM but also to isolated LGA newborns [30,31,32]. In our cohort, the most noticeable difference among high-risk women with no OGTT and a normal OGTT result was extensive weight gain and a more frequent need for operative delivery due to LGA neonate among the normal OGTT group. We may assume that weight gain was the reason for the referral to OGTT. However, we can also speculate that by relating LGA newborns only to GDM, the normal OGTT result could offer false reassurance of a normal pregnancy course and less motivation for weight management after testing.

Women with a GDM diagnosis receive dietary advice or medication (metformin and/or insulin), monitor their blood sugar carefully, and are referred to labor induction more easily. Although, in our study, the number of GDM patients receiving medical treatment was not enough to assess the effect of different treatment modalities on pregnancy outcome, studies have shown the positive effect of GDM treatment on maternal gestational weight gain, perinatal outcome and the possible long term effects on lifestyle changes [33].

However, high-risk women with normal OGTT results should not receive less attention as they are at increased risk of gestational weight gain and a LGA newborn. As a large proportion of these women are overweight, focusing on a healthy diet and exercise have been shown to significantly reduce gestational weight gain [34]. Dodd et al. assessed the addition of metformin to lifestyle interventions; however, they found no complementary benefits from the medication [35]. More targeted prospective studies are needed to determine if quality of diet and additional testing later in pregnancy would add benefits such as timing the delivery and preventing the birth of a LGA among groups of women with GDM risk factors but a normal OGTT result [36,37,38].

A limitation of our study is the small sample size to assess the prevalence of less frequent pregnancy and delivery complications such as preeclampsia, shoulder dystocia and III and IV grade perineal tear in different groups.

## 5. Conclusions

As the number of GDM risk factor carriers is increasing, more women are referred to OGTT and will be diagnosed with GDM with respective pregnancy follow-up. However, we would like to highlight our findings that pregnant women with GDM risk factors are, despite normal OGTT, still at risk of increased weight gain and LGA newborns.

## Figures and Tables

**Table 1:** Maternal characteristics of three datasets ^1^.

Parameter ^2^	I (*n* = 1073)	II (*n* = 2176)	III (*n* = 1772)	*p* Value ^6^		
***Basic characteristics***				*I* vs. *II*	*I* vs. *III*	*II* vs. *III*
Maternal age (years)	28 (20–38)	28 (20–38)	29 (21–38)	n.s	1.9 × 10^−7^	1.2 × 10^−6^
Pre-pregnancy BMI (kg/m^2^)	22.5 (18.0–32.8)	22.3 (18.1–31.5)	22.7 (18.5–33.6)	n.s	n.s	1.9 × 10^−5^
Multiparous ^3^	NA	1190 (54.7%)	1114 (62.9%)	NA	NA	2.2 × 10^−7^
*GDM risk factors*						
Risk factor carriers	467 (43.5%)	1227 (56.4%)	1025 (57.8%)	5.1 × 10^−12^	1.2 × 10^−13^	n.s
Correctly tested among risk factor carriers	291 (62.3%)	702 (57.2%)	787 (76.8%)	n.s	1.5 × 10^−4^	3.5 × 10^−9^
BMI 25–30 kg/m^2^ *n* (% of carriers)	198 (42.4%)	394 (32.1%)	320 (31.2%)	n.s	n.s	n.s
BMI >30 kg/m^2^	92 (19.7%)	185 (15.1%)	210 (20.5%)	n.s	n.s	2.0 × 10^−4^
GDM previously ^3^	13 (2.8%)	26 (2.1%)	28 (2.7%)	n.s	n.s	n.s
Previous baby 4500 g	30 (6.4%)	36 (2.9%)	39 (3.8%)	n.s	n.s	n.s
DM among first degree relatives	78 (16.7%)	224 (18.3%)	161 (15.7%)	n.s	n.s	n.s
PCOS	24 (5.1%)	24 (2.0%)	9 (0.9%)	n.s	3.0 × 10^−5^	n.s
Fasting glycose >5.1 mmol/L	48 (10.3%)	471 (38.4%)	235 (22.9%)	3.3 × 10^−36^	1.6 × 10^−14^	6.0 × 10^−10^
Polyhydramnion	19 (4.1%)	42 (3.4%)	40 (3.9%)	n.s	n.s	n.s
Other ^4^	68 (14.6%)	116 (9.5%)	245 (23.9%)	n.s	6.1 × 10^−10^	3.2 × 10^−20^
*Pregnancy outcome*						
Gestational diabetes	56 (5.2%)	124 (5.7%)	243 (13.7%)	n.s	8.05 × 10^−13^	6.4 × 10^−18^
Preterm birth	50 (4.7%)	110 (5.1%)	87 (4.9%)	n.s	n.s	n.s
Gestational age at delivery (days)	280 (259–287)	281 (258–293)	279 (259–291)	1.38 × 10^−17^	3.69 × 10^−10^	n.s
Birthweight (grams)	3596 (2680–4360)	3569 (2680–4366)	3590 (2660–4302)	n.s	n.s	n.s
Cesarean section	181 (16.9%)	363 (16.7%)	342 (19.3%)	n.s	n.s	n.s
LGA ^5^	202 (18.8%)	341 (15.7%)	286 (16.1%)	n.s	n.s	n.s
LGA + GDM	23 (11.4%)	30 (8.8%)	57 (9.4%)			
SGA ^5^	17 (1.6%)	52 (2.4%)	43 (2.4%)	n.s	n.s	n.s

^1^ I dataset represented women recruited for antenatal care in 2012; II dataset in 2013–2015 and III dataset in 2018. ^2^ Data are given as median (5th–95th percentiles) or number (percentage) when appropriate. Groups were compared using chi-squared test for categorical and Wilcoxon rank-sum test for continuous variables. ^3^ Missing detailed data for number of previous pregnancies in 2012 cohort. ^4^ Risk factors: glycosuria, excessive weight gain (more than 3 kg in 4 weeks) suspicion of LGA fetus in index pregnancy were classified as “other” risk factors. ^5^ For the assignment of a large or small-for-gestational-age (LGA or SGA, respectively) diagnosis, the fetal growth calculator based on INTERGROWTH-21st Project was applied to convert the newborn birthweight into gestational age and sex-adjusted centiles [20]. Newborns were categorized as LGA in cases where the sex-and gestational age adjusted birth centile was more than 95 and SGA in cases where the sex-and gestational age adjusted birth centile was less than 10. centiles ^6^ *p* value was adjusted according to Bonferroni correction for 22 tests and 3 subgroups 0.05/3 × 22 < 7.6 × 10^−4^. DM, diabetes mellitus; GDM, gestational diabetes mellitus; LGA, large for gestational age; NA, not available; n.s, non-significant; PCOS, polycystic ovary syndrome; SGA, small for gestational age.

**Table 2:** Adherence to GDM screening algorithm ^1^ among high-risk women and odds to receive GDM diagnosis.

Risk Factor	Carrier (*n*)	Tested Correctly (*n*/% of Carriers)	GDM (*n*/% of Correctly Tested)	OR (95% CI)	*p*-Value ^3^
BMI 25.0–29.9 kg/m^2^	1385	857 (61.9%)	252 (29.4%)	1.7 (1.4–2.2)	3.2 × 10^−6^
BMI ≥ 30.0 kg/m^2^	482	362 (75.1%)	139 (62.3%)	6.3 (5.0–8.9)	2.9 × 10^−47^
GDM in previous pregnancy	67	59 (88.1%)	36 (62.7%)	12.5 (7.5–20.6)	6.0 × 10^−21^
LGA ^2^ in previous pregnancy	105	88 (83.8%)	32 (38.2%)	4.7 (3.1–7.2)	3.0 × 10^−10^
PCOS	57	41 (71.9%)	14 (53.3%)	3.6 (2.0–6.9)	2.4 × 10^−4^
DM in relatives	463	305 (65.9%)	78 (25.6%)	2.5 (1.9–3.2)	6.1 × 10^−10^
Fasting glycose >5.1 mmol/L	755	436 (57.7%)	175 (40.1%)	5.0 (4.0–6.1)	2.0 × 10^−44^
Polyhydramnion	97	63 (64.9%)	16 (26.2%)	2.1 (1.2–3.6)	1.4 × 10^−2^
Other	431	402 (93.3%)	59 (14.7%)	1.8 (1.3–2.4)	2.1 × 10^−4^

^1^ Schematic representation of GDM screening algorithm is presented in Figure 1. ^2^ For the assignment of large-for-gestational-age (LGA) diagnosis, the fetal growth calculator based on INTERGROWTH-21st Project was applied to convert the newborn birthweight into gestational age and sex-adjusted centiles [20]. Newborn was categorized as LGA in case the sex-and gestational age adjusted birth centile was more than 95. ^3^ *p* value was adjusted according to Bonferroni correction for 9 tests and 2 groups 0.05/18 is 2.7 × 10^−3^. BMI, body mass index; DM, diabetes mellitus; GA, gestational age; GDM, gestational diabetes mellitus; LGA, large for gestational age; OR, odds ratio; PCOS, polycystic ovary syndrome.

**Table 3:** Pregnancy outcome in women allocated into four subgroups according to GDM risk factors and OGTT result.

Outcome ^1^	Low Risk Pregnancies ^5^	High Risk Pregnancies			Pairwise Comparisons
	(Group 1)	No OGTT (Group 2)	OGTT Normal (Group 3)	GDM (Group 4)	Between Groups ^6^ *p* < 7.6 × 10^−4^
Number of women	2302	939	1357	423	
GA at delivery (days)	280 (259–292)	280 (255–292)	280 (260–293)	276 (252–289)	1 vs. 4, 2 vs. 4, 3 vs. 4
Birthweight (grams)	3502 (2644–4233)	3576 (2642–4320)	3705 (2808–4468)	3635 (2695–4430)	1 vs. 2, 1 vs. 3, 1 vs. 4, 2 vs. 3
Birth centile	70.7 (13.7–97.3)	75.7 (18.4–98.2)	82.2 (25.0–99.3)	82.6 (26.5–99.3)	1 vs. 2, 1 vs. 3, 1 vs. 4, 2 vs. 3, 2 vs. 4
LGA ^2^	243 (10.5%)	160 (17.1%)	315 (23.2%)	110 (26.0%)	1 vs. 2, 1 vs. 3, 1 vs. 4, 2 vs. 3, 2 vs. 4
SGA ^2^	70 (3.0%)	15 (1.6%)	23 (1.7%)	4 (0.95%)	n.s
Cesarean section	322 (14.0%)	175 (18.7%)	274 (20.2%)	114 (27.0%)	1 vs. 3, 1 vs. 4; 2 vs. 4
Preterm delivery	104 (4.5%)	55 (5.9%)	58 (4.3%)	27 (6.4%)	n.s
Shoulder dystocia ^3,4^	6/1468 (0.4%)	3/627 (0.5%)	6/883 (0.7%)	1/266 (0.4%)	n.s
Perineal rupture ≥3 grade ^3,4^	14/1468 (1.0%)	3/627 (0.5%)	9/883 (1.0%)	2/266 (0.8%)	n.s
Preeclampsia	23 (1.04%)	23 (2.5%)	25 (1.8%)	11 (2.6%)	1 vs. 2, 1 vs. 3, 1 vs. 4
Gestational hypertension ^4^	20/1699 (1.2%)	26 (3.4%)	50 (4.5%)	25 (6.8%)	1 vs. 4

^1^ Data are given as median (5th–95th percentiles) or number (percentage) when appropriate. ^2^ For the assignment of large or small-for-gestational-age (LGA or SGA, respectively) diagnosis, the fetal growth calculator based on INTERGROWTH-21st Project was applied to convert the newborn birthweight into gestational age and sex-adjusted centiles [20]. Newborn was categorized as LGA in cases where the sex and gestational age adjusted birth centile was more than 95 and SGA in cases where the sex-and gestational age adjusted birth centile was less than 10 centiles. ^3^ Percentage is calculated from vaginal deliveries only. ^4^ Data available for 2013–2015 and 2018 cohorts. ^5^ Low risk pregnancies for GDM were defined as absence of GDM risk factors, for those individuals OGTT is not indicated. High-risk pregnancies for GDM were defined as the presence of any of the following risk factors: BMI > 25 kg/m^2^, GDM or LGA in previous pregnancy, fasting glycose >5.1 mmol/L, PCOS, polyhydramnion, DM in family history, “other” risk factors (glycosuria, excessive weight gain (more than 3 kg in 4 weeks) suspicion of LGA fetus in index pregnancy). Presence of any risk factor is indication for OGTT. ^6^ Wilcoxon rank-sum test was used for continuous variables and Chi2 test for categorical variables, statistical significance level adjusted according to Bonferron correction for 11 parameters and 4 groups 0.05/66 < 7.6 × 10^−4^. DM, diabetes mellitus; GA, gestational age; GDM, gestational diabetes mellitus; LGA, large for gestational age; OGTT, oral glucose tolerance test; PCOS, polycystic ovary syndrome, SGA, small for gestational age.

**Table 4:** Crude and adjusted odds ratios for selected pregnancy outcomes between groups devided according to GDM risk factors and OGTT result.

Outcome	Group	Number of Women	OR (95% CI)	AOR (95% CI)
LGA newborn ^1,2^				
	Low risk	243	1	1
	No OGTT	160	1.8 (1.4–2.2) ***	1.6 (1.2–2.2) ***
	Normal OGTT	315	2.6 (2.1–3.1) ***	2.3 (1.8–3.0) ***
	GDM	110	3.0 (2.3–3.9) ***	2.4 (1.7–3.4) ***
SGA ^2,3^				
	Low risk	70	1	1
	No OGTT	15	0.5 (0.3–0.9) *	0.6 (0.3–1.1)
	Normal OGTT	23	0.5 (0.3–0.9) *	0.6 (0.4–1.0) *
	GDM	4	0.3 (0.1–0.8) *	0.3 (0.1–0.9) *
Preeclampsia ^4^				
	Low risk	23	1	1
	No OGTT	23	2.5 (1.4–4.5) **	1.4 (0.7–2.7)
	Normal OGTT	25	1.9 (1.2–3.3) *	1.1 (0.6–2.1)
	GDM	11	2.6 (1.3–5.5) *	1.3 (0.5–3.1)
Cesarean Section ^5^				
	Low risk	322	1	1
	No OGTT	175	1.4 (1.2–1.7) **	1.2 (0.9–1.5)
	Normal OGTT	274	1.6 (1.3–1.9) ***	1.3 (1.1–1.7) *
	GDM	114	2.3 (1.8–2.9) ***	1.5 (1.1–2.1) *

^1^ Adjusted to previous births, previous LGA baby, BMI, age, cohort and gestational age at delivery. ^2^ For the assignment of large or small-for-gestational-age (LGA or SGA, respectively) diagnosis, the fetal growth calculator based on INTERGROWTH-21st Project was applied to convert the newborn birthweight into gestational age and sex-adjusted centiles [20]. Newborn was categorized as LGA in case the sex-and gestational age adjusted birth centile was more than 95 and SGA in case the sex-and gestational age adjusted birth centile was less than 10 centile. ^3^ Adjusted to cohort, previous births and gestational age at delivery. ^4^ Adjusted to previous births, BMI, and cohort, ^5^ Adjusted to previous births, previous LGA baby, BMI, age, cohort and gestational age at delivery. * *p* < 0.05, ** *p* < 0.005; *** *p* < 0.001. GDM, gestational diabetes mellitus; GH, gestational diabetes; OGTT, oral glycose tolerance test; OR, odds ratio; AOR, adjusted odds ratio.

**Table 5:** Maternal characteristics and pregnancy course among high-risk pregnant women without GDM diagnosis.

	OGTT Normal N = 1357	No OGTT N = 939	*p*-Value ^5^
*Basic characteristics* ^1^			
Age (years)	28 (20–38)	29 (21–39)	n.s
BMI (kg/m^2^)	24.4 (18.7–34.6)	25.3 (18.6–32.9)	n.s
Multiparous ^2^	48.5%	49.3%	n.s
*Risk factorsn* (% of carriers)			
Previous baby 4500 g	60 (4.4%)	13 (1.4%)	1.2 × 10^−4^
GDM previously	25 (1.8%)	4 (0.4%)	n.s
DM among first degree relatives	243 (17.9%)	142 (15.1%)	n.s
PCOS	30 (2.2%)	13 (1.4%)	n.s
Fasting glycose >5.1 mmol/L	267 (19.7%)	311 (33.1%)	7.0 × 10^−14^
Polyhydramnion	47 (3.5%)	38 (4.0%)	n.s
Other	358 (26.4%)	35 (3.7%)	1.3 × 10^−57^
*Pregnancy course and outcome*			
Weight gain (0–23 g.w) (kg)	7 (0–16)	6 (−1–14)	2.2 × 10^−6^
Weight gain (24–42 g.w) (kg)	11 (3.6–22)	10 (2.9–17)	2.0 × 10^−5^
Total weight gain (kg)	17.7 (4–36.5)	15.8 (3–29.6)	3.6 × 10^−5^
Excessive weight gain ^3^	62.4%	53.9%	2.9 × 10^−4^
GA at delivery (days)	280 (260–293)	280 (255–292)	n.s
Male newborn	52.5%	50.0%	n.s
Birthweight (grams)	3705 (2808–4468)	3578 (2642–4320)	5.8 × 10^−9^
LGA ^4^	315 (23.2%)	161 (17.1%)	4.2 × 10^−4^
Birthweight centile	82.2 (25–99.1)	75.7 (18.4–98.2)	5.2 × 10^−9^
Cesarean section	274 (20.2%)	175 (18.6%)	n.s
If LGA (% of Cesarean sections)	87 (31%)	32 (18%)	2.0 × 10^−3^

^1^ Data are given as median (5th–95th percentiles) or number (percentage) when appropriate. Groups were compared using chi-squared test for categorical and Wilcoxon rank-sum test for continuous variables. ^2^ Data not available for 2012 cohort. ^3^ Total weight gain during the pregnancy was considered excessive when it exceeded recommendations by Rasmussen [22]. ^4^ For the assignment of large or small-for-gestational-age (LGA or SGA, respectively) diagnosis, the fetal growth calculator based on INTERGROWTH-21st Project was applied to convert the newborn birthweight into gestational age and sex-adjusted centiles [20]. Newborn was categorized as LGA in cases where the sex-and gestational age adjusted birth centile was more than 95 and SGA in cases where the sex-and gestational age adjusted birth centile was less than 10 centiles. ^5^ *p* value was adjusted for multiple testing according to Bonferroni correction for 2 groups 0.05/21 < 2.4 × 10^−3^. DM, diabetes mellitus; GA, gestational age; g.w, gestational weeks; GDM, gestational diabetes mellitus; LGA, large for gestational age; n.s, non-significant; OGTT, oral glycose tolerance test; PCOS, polycystic ovary syndrome; SGA, small for gestational age.

## Data Availability

The datasets used and/or analyzed during the current study are available from the corresponding author on reasonable request.

## Supplementary Materials

The following supporting information can be downloaded at: www.mdpi.com/article/10.3390/jcm11174953/s1, Table S1: Data acquisition for the study in three time periods. Table S2: Comparison of pregnancy course and outcome in GDM patients according to treatment modalities.

## References

[1] (2019). Hyperglycemia in pregnancy: International Diabetes Federation. IDF Diabetes Atlas.

[2] He X.J., Qin F.Y., Hu C.L., Zhu M., Tian C.Q., Li L. (2015). Is gestational diabetes mellitus an independent risk factor for macrosomia: A meta-analysis?. Arch Gynecol. Obstet..

[3] HAPO Study Cooperative Research Group (2002). The Hyperglycemia and Adverse Pregnancy Outcome (HAPO) Study. Int. J. Gynaecol. Obstet..

[4] Metzger B.E., Lowe L.P., Dyer A.R., Dyer A.R., Trimble E.R., Sheridan B., Hod M., Chen R., Yogev Y., HAPO Study Cooperative Research Group (2008). Hyperglycemia and adverse pregnancy outcomes. N. Engl. J. Med..

[5] Auvinen A.M., Luiro K., Jokelainen J., Järvelä I., Knip M., Auvinen J., Tapanainen J.S. (2020). Type 1 and type 2 diabetes after gestational diabetes: A 23-year cohort study. Diabetologia.

[6] Tobias D.K., Hu F.B., Chavarro J., Rosner B., Mozaffarian D., Zhang C. (2012). Healthful dietary patterns and type 2 diabetes mellitus risk among women with a history of gestational diabetes mellitus. Arch. Intern. Med..

[7] Benhalima K., Mathieu C., Van Assche A., Damm P., Devlieger R., Mahmood T., Dunne F. (2016). Survey by the European Board and College of Obstetrics and Gynaecology on screening for gestational diabetes in Europe. Eur. J. Obstet. Gynecol. Reprod. Biol..

[8] Minschart C., Beunen K., Benhalima K. (2021). An Update on Screening Strategies for Gestational Diabetes Mellitus: A Narrative Review. Diabetes Metab. Syndr. Obes..

[9] International Association of Diabetes and Pregnancy Study Groups Consensus Panel (2010). International association of diabetes and pregnancy study groups recommendations on the diagnosis and classification of hyperglycemia in pregnancy. Diabetes Care.

[10] Benhalima K., Van Crombrugge P., Moyson C., Verhaeghe J., Vandeginste S., Verlaenen H., Vercammen C., Maes T., Dufraimont E., De Block C. (2019). Risk factor screening for gestational diabetes mellitus based on the 2013 WHO criteria. Eur. J. Endocrinol..

[11] Diabetes in Pregnancy: Management from Preconception to the Postnatal Period: NICE Guideline. Updated: 16 December. https://www.nice.org.uk/guidance/ng3/chapter/Recommendations.

[12] Yamamoto J.M., Kellett J.E., Balsells M., García-Patterson A., Hadar E., Solà I., Gich I., van der Beek E.M., Castañeda-Gutiérrez E., Heinonen S. (2018). Gestational Diabetes Mellitus and Diet: A Systematic Review and Meta-analysis of Randomized Controlled Trials Examining the Impact of Modified Dietary Interventions on Maternal Glucose Control and Neonatal Birth Weight. Diabetes Care.

[13] Thayer S.M., Lo J.O., Caughey A.B. (2020). Gestational Diabetes: Importance of Follow-up Screening for the Benefit of Long-term Health. Obstet. Gynecol. Clin. N. Am..

[14] Avalos G.E., Owens L.A., Dunne F., ATLANTIC DIP Collaborators (2013). Applying current screening tools for gestational diabetes mellitus to a European population: Is it time for change?. Diabetes Care.

[15] Kirss A., Lauren L., Rohejärv M., Rull K. (2015). Gestatsioonidiabeet: Riskitegurid, esinemissagedus, perinataalne tulem ja sõeluuringu vastavus juhendile Tartu Ülikooli Kliinikumi naistekliinikus ajavahemikul 01.01.2012–19.06. East Arst.

[16] Ratnik K., Rull K., Hanson E., Kisand K., Laan M. (2020). Single-Tube Multimarker Assay for Estimating the Risk to Develop Preeclampsia. J. Appl. Lab. Med..

[17] Kikas T., Inno R., Ratnik K., Rull K., Laan M. (2020). C-allele of rs4769613 Near FLT1 Represents a High-Confidence Placental Risk Factor for Preeclampsia. Hypertension.

[18] Hanson E., Rull K., Ratnik K., Vaas P., Teesalu P., Laan M. (2022). Value of soluble fms-like tyrosine kinase-1/placental growth factor test in third trimester of pregnancy for predicting preeclampsia in asymptomatic women. J. Perinat. Med..

[19] Vaas P., Rull K., Põllumaa S., Kirss A., Meigas D. Guideline for Antenatal Care (Raseduse Jälgimise Juhend). Estonian Gynaecologists Society 2011; vEstonian. https://www.ens.ee/ravijuhendid.

[20] Villar J., Papageorghiou A.T., Pang R., Ohuma E.O., Ismail L.C., Barros F. (2014). The likeness of fetal growth and new-born size across non-isolated populations in the INTERGROWTH-21st Project: The Fetal Growth Longitudinal Study and Newborn Cross-Sectional Study. Lancet.

[21] Brown M.A., Magee L.A., Kenny L.C., Karumanchi S.A., McCarthy F.P., Saito S., Hall D.R., Warren C.E., Adoyi G., Ishaku S. (2018). Hypertensive Disorders of Pregnancy: ISSHP Classification, Diagnosis, and Management Recommendations for International Practice. Hypertension.

[22] Rasmussen K.M., Abrams B., Bodnar L.M., Butte N.F., Catalano P.M., Maria Siega-Riz A. (2010). Recommendations for weight gain during pregnancy in the context of the obesity epidemic. Obstet. Gynecol..

[23] Muin D.A., Pfeifer B., Helmer H., Oberaigner W., Leitner H., Kiss H., Neururer S. (2022). Universal gestational diabetes screening and antepartum stillbirth rates in Austria-A population-based study. Acta Obstet. Gynecol. Scand..

[24] Koivunen S., Viljakainen M., Männistö T., Gissler M., Pouta A., Kaaja R., Eriksson J., Laivuori H., Kajantie E., Vääräsmäki M. (2020). Pregnancy outcomes according to the definition of gestational diabetes. PLoS ONE.

[25] Mohr Sasson A., Shats M., Goichberg Z., Mazaki-Tovi S., Morag I., Hendler I. (2021). Oral glucose tolerance test for suspected late onset gestational diabetes. J. Matern. Fetal. Neonatal. Med..

[26] Mo X., Gai Tobe R., Takahashi Y., Arata N., Liabsuetrakul T., Nakayama T., Mori R. (2021). Economic Evaluations of Gestational Diabetes Mellitus Screening: A Systematic Review. J. Epidemiol..

[27] Xiang A.H., Kjos S.L., Takayanagi M., Trigo E., Buchanan T.A. (2010). Detailed physiological characterization of the development of type 2 diabetes in Hispanic women with prior gestational diabetes mellitus. Diabetes.

[28] Tuomilehto J., Lindstrom J., Eriksson J.G., Valle T.T., Hämäläinen H., Ilanne-Parikka P., Keinänen-Kiukaanniemi S., Laakso M., Louheranta A., Rastas M. (2001). Prevention of type 2 diabetes mellitus by changes in lifestyle among subjects with impaired glucose tolerance. N. Engl. J. Med..

[29] Ratner R.E., Christophi C.A., Metzger B.E., Dabelea D., Bennett P.H., Pi-Sunyer X., Fowler S., Kahn S.E., Diabetes Prevention Program Research Group (2008). Prevention of diabetes in women with a history of gestational diabetes: Effects of metformin and lifestyle intervention. J. Clin. Endocrinol. Metab..

[30] Zhao R., Xu L., Wu M.L., Huang S.H., Cao X.J. (2018). Maternal pre-pregnancy body mass index, gestational weight gain influence birth weight. Women Birth.

[31] Usta A., Usta C.S., Yildiz A., Ozcaglayan R., Dalkiran E.S., Savkli A., Taskiran M. (2017). Frequency of fetal macrosomia and the associated risk factors in pregnancies without gestational diabetes mellitus. Pan Afr. Med. J..

[32] Lin L.H., Lin J., Yan J.Y. (2022). Interactive Affection of Pre-Pregnancy Overweight or Obesity, Excessive Gestational Weight Gain and Glucose Tolerance Test Characteristics on Adverse Pregnancy Outcomes Among Women with Gestational Diabetes Mellitus. Front. Endocrinol..

[33] Rasmussen L., Poulsen C.W., Kampmann U., Smedegaard S.B., Ovesen P.G., Fuglsang J. (2020). Diet and Healthy Lifestyle in the Management of Gestational Diabetes Mellitus. Nutrients.

[34] Peaceman A.M., Clifton R.G., Phelan S., Gallagher D., Evans M., Redman L.M., Knowler W.C., Joshipura K., Haire-Joshu D., Yanovski S.Z. (2018). Lifestyle Interventions Limit Gestational Weight Gain in Women with Overweight or Obesity: LIFE-Moms Prospective Meta-Analysis. Obesity.

[35] Dodd J.M., Louise J., Deussen A.R., Grivell R.M., Dekker G., McPhee A.J., Hague W. (2019). Effect of metformin in addition to dietary and lifestyle advice for pregnant women who are overweight or obese: The GRoW randomised, double-blind, placebo-controlled trial. Lancet Diabetes Endocrinol..

[36] Zhu Y., Hedderson M.M., Sridhar S., Xu F., Feng J., Ferrara A. (2019). Poor diet quality in pregnancy is associated with increased risk of excess fetal growth: A prospective multi-racial/ethnic cohort study. Int. J. Epidemiol..

[37] Han S., Crowther C.A., Middleton P. (2012). Interventions for pregnant women with hyperglycaemia not meeting gestational diabetes and type 2 diabetes diagnostic criteria. Cochrane Database Syst. Rev..

[38] Munda A., Starčič Erjavec M., Molan K., Ambrožič Avguštin J., Žgur-Bertok D., Pongrac Barlovič D. (2019). Association between pre-pregnancy body weight and dietary pattern with large-for-gestational-age infants in gestational diabetes. Diabetol. Metab. Syndr..

