# Supplementary material for: Screening of Gestational Diabetes and Its Risk Factors: Pregnancy Outcome of Women with Gestational Diabetes Risk Factors According to Glycose Tolerance Test Results
Source: J Clin Med. 2022 Aug 23;11(17):4953. doi: 10.3390/jcm11174953 (PMC9456276; doi:10.3390/jcm11174953)
Supplement: Supplementary file 1 [file jcm-11-04953-s001.zip › jcm-1809057-supplementary.pdf]

Table S1. Data acquisation for the study in three time period.

Parameter	Time Period		
	2012	2013–2015	2018
Basic characteristics			
Maternal age	Calculation based ID-code	Calculation based ID-code	Calculation based ID-code
Pre-pregnancy BMI	Documented by midwife	Patient reported and hospital database	hospital database
Number of previous deliveries	NA	Patient reported and hospital database	hospital database
GDM risk factors			
GDM previously	Documented by midwife	Patient reported and hospital database	hospital database
Previous baby 4500g	Documented by midwife	Patient reported and hospital database	hospital database
DM among first degree relatives	Documented by midwife	Patient reported and hospital database	hospital database
PCOS	Documented by midwife	Patient reported and hospital database	hospital database
Fasting glycose >5.1mmol/L	Laboratory database	Laboratory database	Laboratory database
Polyhydramnion	Documented by midwife	hospital database	hospital database
Glucosuria	Documented by midwife	hospital database	hospital database
Gestational weight gain	NA	Patient reported and hospital database	hospital database
Excessive weight gain	Documented by midwife	Calculation based weight gain and pre-pregnancy BMI	Calculation based weight gain and pre-pregnancy BMI
Suspicion to fetal macrosomia	Documented by midwife	hospital database	hospital database
Outcome of pregnancy			
GDM	Laboratory database	Laboratory and hospital database	Laboratory and hospital database
Hypertensive disorders	NA	hospital database	hospital database
Gestational age at delivery	hospital database	hospital database	hospital database
Delivery mode	hospital database	hospital database	hospital database
Birth parameters	hospital database	hospital database	hospital database
Fetal macrosomia			
Shoulder dystocia	hospital database	hospital database	hospital database
Perineal tear ≥3 grade	hospital database	hospital database	hospital database

Midwives completed GDM risk assessment check-list (pre-gestational BMI, GDM and/or of birth baby >4500g during previous pregnancy, DM among first-degree relatives, previous PCOS, abnormal fasting/random blood glycose, excessive weight gain, glucosuria, macrosomia or polyhydramnion detected at ultrasound scan) twice during the pregnancy. Detailed information about previous pregnancies and total weight gain during pregnancy is not available for this cohort. Patient reported data originate from questionairres filled by themselves during the pregnancy. Data were revised and complented when discrepancies between patient's report and medical data were detected. Data from hospital database derived directly from electronic records and no manual data entry was performed.

Table S2. Pregnancy course and outcome in women with GDM diagnosi.

Outcome [1]	GDM in Total *n* = 423	Only Diet [2] *n* = 285	Diet and Metformin and/or Insulin [2] *n* = 82	Comparison between Treatment Groups *p*-Value [3]
Maternal age (years)	31 (22–39)	30 (21–39)	30 (23–38)	n.s
Pre-pregnancy BMI (kg/m^2)	26.4(19.2–37.9)	25.7 (19.1–36.5)	29.1 (20.2–42.2)	2.7×10^{-3}
Weight gain (0–23 g.w) (kg)	5.0 (−3.0–13)	5.0 (−2.0–13)	6.0 (−4.0–16)	n.s
Weight gain (24–42 g.w) (kg)	8.0 (1.0–17)	7.8 (1.6–17)	8.0 (−2.0–19)	n.s
Total weight gain (kg)	13 (−0.4–29.1)	13 (1.5–29)	13 (−5.2–33.4)	n.s
Gestational age at delivery (days)	276 (252–289)	277 (257–290)	274.5 (259–288)	**1.3×10^{-3}**
Birthweight (grams)	3635 (2695–4430)	3602 (2780–4356)	3573 (2780–4564)	n.s
Birth centile	82.6 (26.5–99.3)	79.9(26.5–98.8)	82.3 (19.7–99.8)	n.s
LGA [4]	110 (26.0%)	61 (21.4%)	26 (31.7%)	5.3×10^{-2}
SGA [4]	4 (0.95%)	2 (0.7%)	2 (2.4%)	n.s
Cesarean section	114 (27.0%)	75 (26.3%)	26 (31.7%)	n.s
Preterm delivery	27 (6.4%)	17 (6.2%)	3 (3.8%)	n.s
Shoulder dystocia [5]	1/266 (0.4%)[2]	0/210 (0%)	1/56 (1.8%)	n.s
Perineal rupture ≥3 grade [5]	2/266 (0.8%)[2]	1/210 (0.5%)	1/56 (1.8%)	n.s
Preeclampsia	11 (2.6%)	6 (2.1%)	4 (4.9%)	n.s
Gestational hypertension	25 (6.8%)[2]	19 (6.7%)	6 (7.3%)	n.s

[1] Data are given as median (5th–95th percentiles) or number (percentage) when appropriate. [2] Detailed data about GDM treatment was available for 2013–2015 and 2018 cohorts, and not for 56 women representing I dataset, recruited for antenatal care in 2012. [3] Wilcoxon rank-sum test was used for continuous variables and Chi2 test for categorical variables, statistical significance level adjusted according to Bonferron correction for 16 parametes and 2 groups $0.05/32 < 1.6 \times 10^{-3}$, parameters exceeding the level are shown in bold. [4] For the assignment of large or small-for-gestational-age (LGA or SGA, respectively) diagnosis, the fetal growth calculator based on INTERGROWTH-21st Project was applied to convert the newborn birthweight into gestational age and sex-adjusted centiles (20). Newborn was categorized as LGA in case the sex-and gestational age adjusted birth centile was more than 95 and SGA in case the sex-and gestational age adjusted birth centile was less than 10 centiles. [5] Percentage is calculated from vaginal deliveries only. BMI, body mass index; GDM gestational diabetes; g.w, gestational weeks.
